# Highlight selection of radiochemistry and radiopharmacy developments by editorial board

**DOI:** 10.1186/s41181-025-00387-y

**Published:** 2025-10-03

**Authors:** Jun Toyohara, Philipp H. Elsinga, Sergio Todde, Xiang-Guo Li, Silvio Aime, Wictor C. Szymanski, Nick van der Meulen, Frederik Cleeren, Ralf Schirrmacher, Hua Yang, Eduardo Savio, Naoual Bentaleb, Marcela Zubillaga, Ivis F. Chaple Gore, Mickaël Bourgeois, Archana Mukherjee, Juan Pellico, Klaus Kopka

**Affiliations:** 1 Tokyo Metropolitan Institute for Geriatrics and Gerontology, Tokyo, Japan; 2 University of Groningen, Groningen, The Netherlands; 3 University of Milano-Bicocca, Monza, Italy; 4 Turku PET Centre, University of Turku, Turku, Finland; 5 IRCCS SDN SynLab, Naples, Italy; 6 Paul Scherrer Institute, Villigen, Switzerland; 7 Katholieke Universiteit, Leuven, Belgium; 8 Medical Isotope and Cyclotron Facility, Affiliated member Chemistry and Pharmacy, University of Alberta, Cross Cancer Institute, University of Alberta, Edmonton, AB Canada; 9 TRIUMF, Vancouver, BC Canada; 10 Uruguayan Center for Molecular Imaging (CUDIM), Montevideo, Uruguay; 11 National Center for Nuclear Energy, Science and Technology (CNESTEN), Rabat, Morocco; 12 University of Buenos Aires, Buenos Aires, Argentina; 13 University of Tennessee, Knoxville, USA; 14 Nantes University Hospital - Nuclear Medicine Department, Nantes, France; 15 Faculty of Pharmacy - Galenic/Biophysic Department, ARRONAX Cyclotron - Radiopharmacy Unit, France Nantes University, Nantes, France; 16 Bhabha Atomic Research Centre & Homi Bhabha National Institute, Mumbai, India; 17 Institute of Materials Science of Barcelona (ICMAB-CSIC), Barcelona, Spain; 18 Institute of Radiopharmaceutical Cancer Research, Faculty of Chemistry and Food Chemistry, School of Science, Helmholtz-Zentrum Dresden- Rossendorf (HZDR), Technical University Dresden (TUD), Dresden, Germany

**Keywords:** Highlight articles, Radiochemistry, Radiopharmacy, Radiopharmaceutical sciences, Nuclear medicine

## Abstract

**Background:**

The Editorial Board of EJNMMI Radiopharmacy and Chemistry releases a biannual highlight commentary to update the readership on trends in the field of radiopharmaceutical development and application of radiopharmaceuticals.

**Main Body:**

This selection of highlights provides commentary on 18 different topics selected by each co-authoring Editorial Board member addressing a variety of aspects ranging from novel radiochemistry to first-in-human application of novel radiopharmaceuticals.

**Conclusion:**

Trends in radiochemistry and radiopharmacy are highlighted. Hot topics cover the entire scope of EJNMMI Radiopharmacy and Chemistry, demonstrating the progress in the research field in many aspects.

## Background

Each individual co-authoring member of the Editorial Board has selected to highlight an article that has appeared in the radiochemistry, radiopharmacy and imaging agent literature during the period January-June 2025. The aim of this collaborative initiative is to create a biyearly overview for the readers summarizing the latest trends and hot topics in the field.

## Selected highlight articles

### A simplified radiosynthetic approach to ^18^F-labelling BODIPY dyes using indium salts as ideal Lewis acid mediators

By Ralf Schirrmacher

**Fig. 1:**
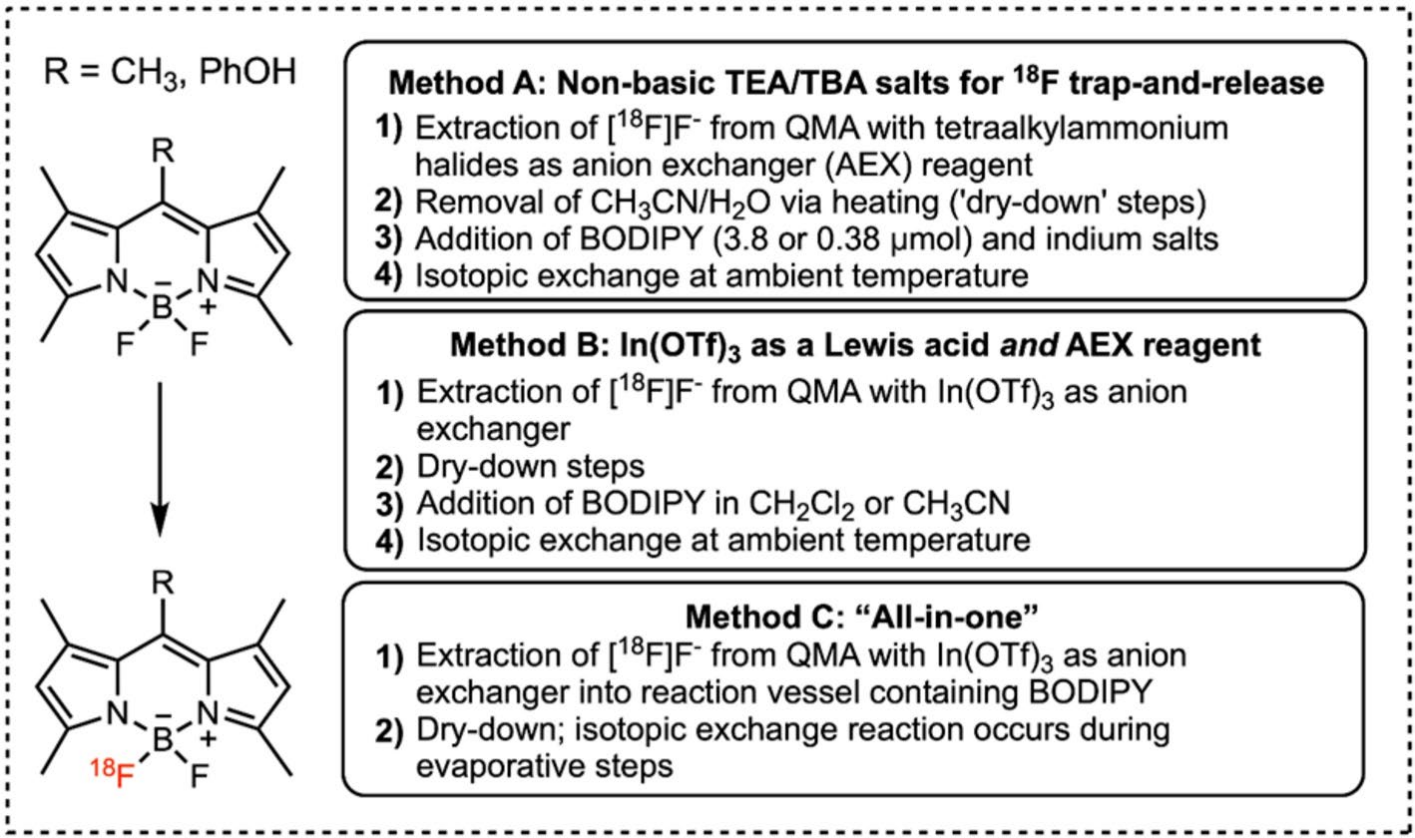
Overview of methods to prepare ^18^F-labelled BODIPY derivatives. Figure reproduced from (Inkster et al. 2025) under Creative Commons CC-BY license

There is always room for improvement. Indium Salt–Mediated ¹⁸F-Labeling based on isotopic exchange of BODIPY Dyes for PET Imaging has been jointly reported by the Inkster, Phenix and Price groups (Inkster et al. 2025). Indium salts efficiently mediate ¹⁸F/¹⁹F isotopic exchange in meso-methyl- or meso-phenol-substituted BODIPY dyes using dried tetraalkylammonium [¹⁸F]fluoride (Fig. 1). Aqueous [¹⁸F]F⁻ was trapped on QMA cartridges and eluted with non-basic chloride or triflate, yielding 80–98% decay-corrected (DC) activity retention after evaporation at 110 °C—despite the absence of traditional carbonate bases. In CH₂Cl₂ or CH₃CN, the method furnished ¹⁸F-labeled BODIPYs in 47–60% DC radiochemical yields after solid-phase extraction. In(OTf)₃ served dual roles in [¹⁸F]F⁻ elution and Lewis acid activation but led to higher [¹⁸F]HF loss (60 ± 8% DC retention post-evaporation). A streamlined one-pot route using In(OTf)₃-eluted [¹⁸F]F⁻ and acetonitrile–water dye solutions followed by C18 SPE achieved radiochemical yields of 27–39% in 50–73 min—suitable for automation.

This protocol offers a simplified, SnCl₄-free approach for converting BODIPY dyes into dual fluorescence and PET imaging agents and might even provide a general improvement for isotopic exchange reactions involving B-F containing molecules and beyond.

### Efforts towards standardizing synthesis and quality control methods for [^225^Ac]Ac-PSMA-617 to fully harness the potential of actinium-225

By Naoual Bentaleb

Prostate-specific membrane antigen (PSMA) has emerged as an ideal biomarker for targeted radionuclide therapy (TRT) and imaging in nuclear medicine, particularly for diagnosing and treating prostate cancer. Over the past decade, numerous PSMA-targeting radiopharmaceuticals have been developed, leveraging different radionuclides for diagnostic and therapeutic applications.

For therapeutic applications, both α- and β^−^-emitting radionuclides can be used. Lutetium-177 [^177^Lu], a β^−^-emitter, is widely used for TRT. However, up to 30% of patients with metastatic castration-resistant prostate cancer treated by [^177^Lu]Lu-PSMA therapy do not respond (Feuerecker et al. 2023). To overcome this radioresistance, α-emitters like ^225^Ac combined with PSMA-617 showed promising results in patients who progressed after [^177^Lu]Lu-PSMA-617 therapy (Kratochwil et al. 2016, 2017). This is attributed to the ideal properties of ^225^Ac, including half-life, high cytotoxicity and short path length.

Although significant clinical results have been achieved using [^225^Ac]Ac-PSMA-617, challenges persist, particularly in standardizing synthesis and quality control methods to meet pharmacopoeia standards.

The article titled “[^225^Ac]Ac-PSMA-617 production method: development of an efficient and reproducible radiolabelling process for establishing a clinical routine production” (Aurilio et al. 2025) focuses on optimizing the radiolabeling of PSMA-617 with ^225^Ac.

The study identified the optimal conditions for a reproducible method for [^225^Ac]Ac-PSMA-617 clinical production. These conditions involve using 100 µg of peptide (PSMA-617) in gentisic acid buffer, without the aid of the stabilizing agent. This method achieved a radiochemical purity (RCP) greater than 95% and maintained adequate stability, around 90%, at 24 h. Challenges remain, particularly in the development of an accurate quality control method based on direct α-particle detection instead of indirect method relying on γ-emissions which may limit accuracy due to ^225^Ac’s decay products interfering with measurements and recoil effects. The authors acknowledge the need for alternative chelators to enhance stability as DOTA may not be optimal for radiolabeling of PSMA-617 with ^225^Ac.

### To be or not to be. That Is the question

By Marcela Zubillaga

Downregulation or upregulation? This is the question implicitly raised by the authors who report the production and validation of [¹³N]ammonia under Academic Good Manufacturing Practice (a-GMP) conditions (Tomiyoshi et al. 2025).

While traditional GMP frameworks—such as those defined by EMA, FDA, or PIC/S—were designed for large-scale industrial production with extended distribution chains, the a-GMP approach aligns more closely with the operational needs of clinical institutions. It offers a risk-based, proportionate model that ensures patient and staff safety without the infrastructure burden of industrial compliance.

This work underscores a key regulatory dilemma: when stringent industrial GMP requirements are applied indiscriminately to hospital radiopharmacies, they may inadvertently restrict access to essential radiopharmaceuticals, particularly those with short half-lives. As highlighted in a previous EJNMMI Radiopharm Chem highlight commentary, Perk discussed the need of a critical view from regulatory authorities and thorough risk assessment that must be considered facilitating the access to radiopharmaceuticals (Kiss et al. 2023).

**Fig. 2:**
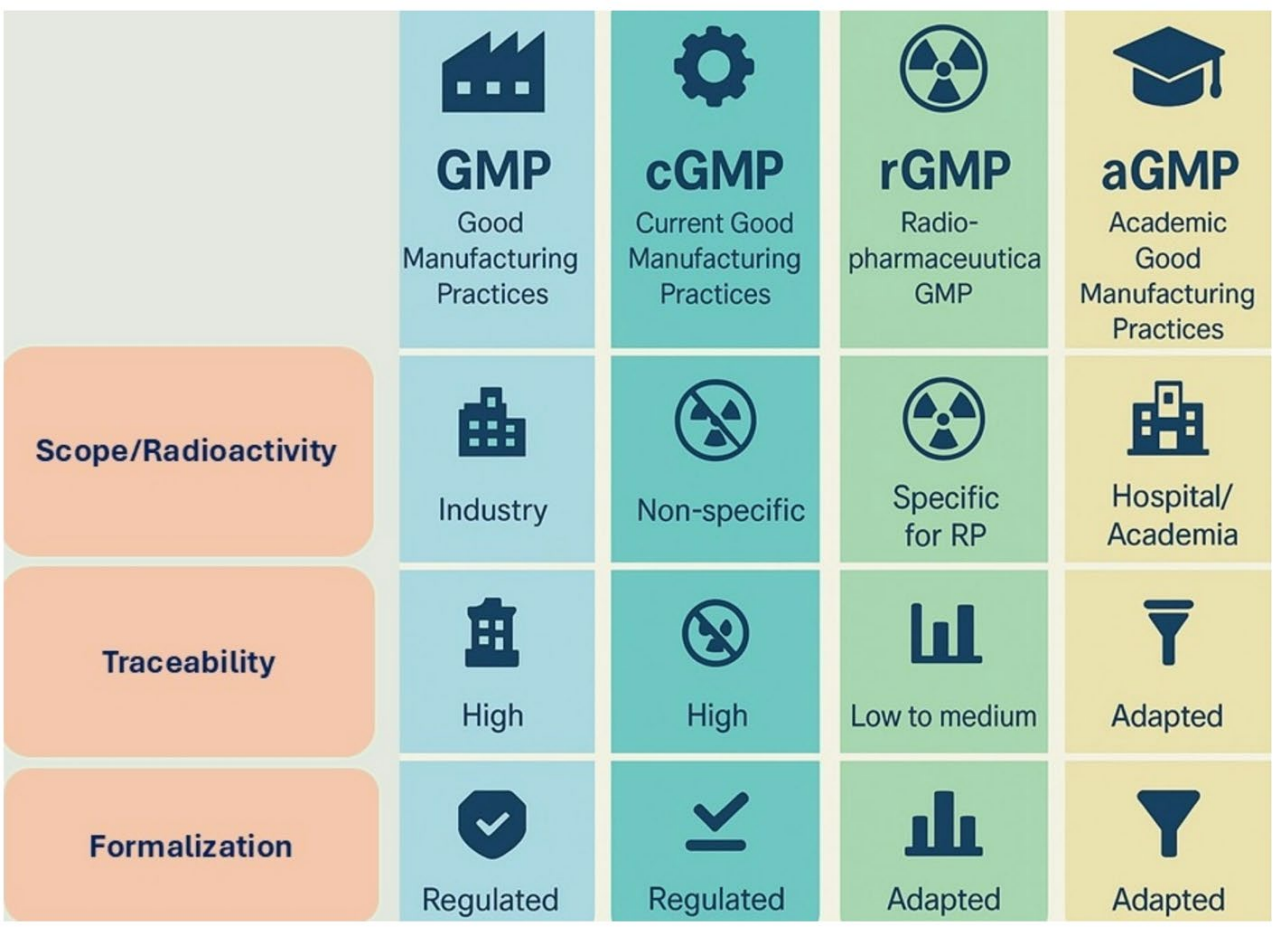
Schematic overview of different GMP frameworks

This study raises several key questions regarding the role of regulatory authorities in verifying and applying different GMP frameworks (Fig. 2). What level of oversight do these agencies assume when determining which guidance should be followed: GMP, c-GMP, r-GMP, or a-GMP? While all models aim to ensure quality and safety in the production and control of radiopharmaceuticals, they differ in how they address critical elements such as risk-based validation, process proportionality, and the availability of certified reference standards. This underscores the need for a harmonized regulatory approach that acknowledges the unique conditions of on-site clinical use. The inclusion of affordable, internationally traceable reference standards remains a major gap, directly impacting the capacity of clinical centers to ensure product quality. In this context, regulatory agencies must go beyond inspection and enforcement: they must actively support the development of flexible, context-sensitive guidelines that promote equitable access to safe and effective radiopharmaceuticals.

### Pretargeting strategies: driving the future of radioimmunotherapy

By Archana Mukherjee.

There has been a resurgence of interest in the development of radioimmunotherapeutic agents that exploit the high affinity and specificity of antibodies or antibody-based vectors, particularly for delivering high-energy β^-^ and α emitting radionuclides. Despite significant progress, these agents have not yet demonstrated the desired therapeutic efficacy in preclinical models to support clinical translation. To address this challenge, pretargeting strategies have been explored. These involve a two-step approach in which a highly specific antibody or targeting vector is first administered to bind the tumor, followed by a radiolabeled carrier with high affinity for the prelocalized antibody. This multi-step method has been shown to enhance the therapeutic index while minimizing systemic toxicity and off-target effects. Cheal et al. have provided a comprehensive review identifying pretargeting as a promising path forward in radioimmunotherapy. Their review discusses emerging approaches, including those based on bioorthogonal chemistry and novel protein scaffolds such as Affibody molecules (Cheal et al. 2022). In a recent study, the same group described a three-step DOTA-PRIT (pretargeted radioimmunotherapy) regimen designed for the targeted treatment of colorectal cancer (Le Roux, 2025). This strategy utilizes a bispecific antibody (BsAb) that recognizes both the tumor-associated glycoprotein A33 (GPA33) and DOTA. Following BsAb localization and subsequent clearance from circulation using a clearing agent, a bivalent ¹⁷⁷Lu-labeled radiohapten, [¹⁷⁷Lu]Lu-Gemini, is administered to enhance tumor uptake and retention. The approach was evaluated in orthotopic colorectal cancer liver metastasis models. The bivalent radiohapten demonstrated superior tumor targeting and prolonged intratumoral retention compared to its monovalent counterpart, along with favorable therapeutic indices for blood and kidney. These findings suggest that such pretargeting strategies may significantly improve the efficacy of antibody-based radiotherapeutics and facilitate their successful clinical translation.

### Production of ^67^Cu at a biomedical cyclotron via ^70^Zn(p,α)^67^Cu reaction and its evaluation in a preclinical study using small animal SPECT/CT

By Ivis F. Chaple Gore.

Radioisotope availability continues to be the predominant challenge impacting global research endeavors in theranostic radiopharmaceutical development. Radiocopper is an excellent option for a theranostic approach towards many types of cancers, particularly because of the possibilities of using ^64^Cu (t_½_ = 12.7 h, β+ = 17%) for positron emission tomography imaging and ^67^Cu ((t_½_ = 61.8 h, β- = 100%) for targeted beta therapy and single photon emission computed tomography imaging. The implementation of the therapeutic radionuclide (^67^Cu) has been hindered due to the shortage in access of this radionuclide for research purposes. The cited article (Søndergaard et al. 2025) does an excellent job in demonstrating that lower energy cyclotrons can play a pivotal role in increasing access to research quantities of ^67^Cu, enabling enhanced research and development of next generation radiopharmaceuticals. While challenges still exist in the targetry, separations radiochemistry, and recycling of the enriched Zn target material, increased efforts should be devoted to overcoming these challenges for increased access to accelerator-produced ^67^Cu.

### Simplification of dosimetry determination in patient treatment?

By Nick van der Meulen.

The use of dosimetry is becoming increasingly important in nuclear medicine, particularly with the introduction of new radiopharmaceuticals for use in theranostics. Currently, this is being performed by taking images using three timepoints – regarded as time consuming and expensive. While there have been proposals and suggestions about single timepoint (STP) determination, several discussions have questioned the accuracy of this determination.

An instant single imaging (iSTP) timepoint using pretherapy patient data for the entire treatment was proposed in a recent paper (Gomes et al. 2025). The machine-learning-based model was developed by incorporating PET imaging quantification, clinical features and the corresponding effective half-life (T_eff_) of the target organ in question. They used this data to train their machine-learning model to predict subsequent cycles.

The authors demonstrated that their iSTP method significantly outperformed STP at the 2-hour timepoint, while later timepoints were still relatively comparable to STP methods. STP appear to have shown limitations in accurately assessing dosimetry at early timepoints post injection. The authors have indicated targeting iSTP application for early timepoints, optimally within the first two days after injection, which is generally more practical for dosimetry implementation.

The authors’ preliminary findings indicate the potential of their proposed iSTP method, towards the simplification of future routine clinical dosimetry implementation. While there is optimism using this method, it is clear that more in-depth studies are necessary.

### Roadmap for PET tracer development for imaging efflux transporters at the blood-brain barrier

By Xiang-Guo Li.

Blood-brain barrier (BBB) is a physical, functional and structural membrane between blood and brain, which governs the efficiency of drug penetration into the brain. At the BBB, there are dedicated transporters for influx and efflux of drugs, metabolites, and biological molecules. Efflux transporters have determining roles for the concentration level that a drug can reach in the brain. Functional changes of efflux transporters have been associated with neurological diseases and drug resistance. PET is a powerful tool for in vivo imaging of the functional changes of efflux transporters and predicting bioavailability of new drug candidates, but better radiotracers are needed. In a recent article (Tournier & Langer 2025), preclinical and clinical evidence on PET imaging of efflux transporters during the last two decades have been critically analyzed, based on which desirable features and criteria of next generation radiotracers have been described. This is a valuable roadmap not only for the development of new radiotracers, but also for new therapeutics for the same targets. The availability of novel radiotracers for PET imaging will enhance our understanding of efflux transporters in disease and health. Similar roadmaps and guidelines would be beneficial for radiotracer development for other targets.

### Mitochondria-tropic radioconjugates to enhance the therapeutic potential of terbium-161

By Juan Pellico.

In this work a very interesting approach is described to enhance targeted radionuclide therapy (TRT) for prostate cancer by using terbium-161, a radionuclide that emits Auger and conversion electrons and holds strong therapeutic potential, despite its limited use in TRT to date (Santos et al. 2025). The team developed two radioconjugates that combine a PSMA-targeting vector with a mitochondria-localizing triphenylphosphonium (TPP) group: [^161^Tb]Tb-TPP-PSMA and [^161^Tb]Tb-TPP-G3-PSMA. Both compounds were synthesized with high radiochemical yield and demonstrated good stability. In vitro experiments in PSMA-positive prostate cancer cells showed efficient uptake, significant mitochondrial localization, and increased DNA damage when compared to the conventional [^161^Tb]Tb-PSMA-617 compound. The G3 variant, which includes a cleavable linker sensitive to cathepsin B, further improved mitochondrial delivery. Clonogenic assays confirmed that the TPP-containing compounds were more cytotoxic than the standard agent. In vivo SPECT/CT imaging in mice bearing PSMA-positive and PSMA-negative tumors confirmed specific tumor uptake, favorable pharmacokinetics, and efficient clearance from non-target tissues. Overall, the findings suggest that dual targeting of PSMA and mitochondria can enhance the therapeutic effect of ^161^Tb-based agents without compromising specificity or safety. In my opinion, this work is highly relevant as it opens new opportunities for targeted radionuclide therapy with ^161^Tb by exploiting intracellular targeting strategies.

### Expanding the chemical space of PET tracers: the case for labeling difluoromethylalkyl groups

By Wiktor Szymanski.

The widespread application of PET imaging relies on the availability of radiolabeled tracers which, in turn, hinges on our ability to introduce radionuclides (for example the often-used fluorine-18) into targeting molecules in an efficient and fast manner. In that respect, the labeling of geminal difluoroalkanes is a sought-after methodology, since difluoromethyl groups act as metabolically stable bioisosteres of hydroxy, thio and amine groups, due to their ability of acting as hydrogen bond donors.

**Fig. 3:**
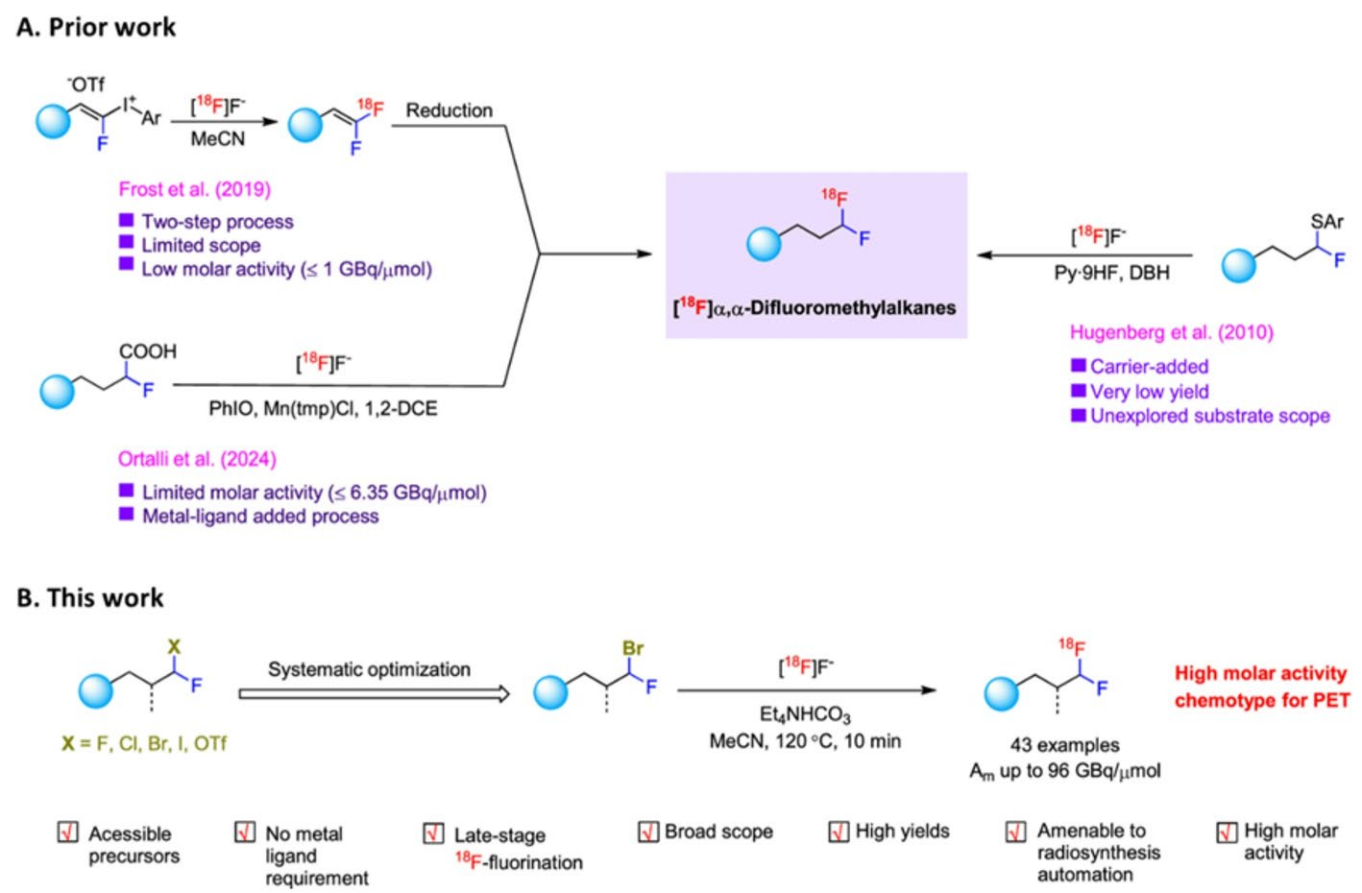
Synthetic approaches to the [^18^F]CHF_2_ group. (**A**) Limitations of the existing methods; (**B**) The method introduced by Zhao et al. with the list of associated advantages. Figure reproduced from Zhao et al.^,^ 2025 under the CC BY license

While several synthetic approaches have been established (Fig. 3A), they all suffer from certain limitations, including the limited scope, low molar activity and low yields. Conversely, in a recent report (Zhao et al. 2025), this synthetic challenge was approached through a simple nucleophilic substitution of mono-fluoroalkanes bearing a leaving group in the α-position. While seemingly obvious, this approach was still not straightforward, due to several challenges. Firstly, it was expected that the release of cold fluoride from the precursor molecule could compromise molar activity of the product. Secondly, the presence of fluoride could impede the substitution reaction, while the presence of the leaving group could lead to elimination product.

Altogether, those challenges necessitated a careful optimization of reaction conditions, leading to the labelling system displayed in Fig. 3B. Under those conditions, an impressive substrate scope of 28 model compounds and 15 biologically relevant molecules could be labelled with fluorine-18. The reaction lent itself also to automation, providing several products with 0.8–13 GBq of products in 60–70 min reaction time.

Altogether, the high efficiency, compatibility with late-stage functionalization and automation render this approach a valuable addition to the repertoire of modern ^18^F-labeling methods.

### Hsp90 binding tracer as a candidate for protein homeostasis imaging

By Jun Toyohara.

Heat shock protein 90 (Hsp90) plays a crucial role in maintaining cellular proteostasis by stabilizing client proteins such as β-amyloid, phosphorylated tau, and α-synuclein. Accordingly, Hsp90 has been implicated in neurodegenerative proteinopathies, and numerous therapeutic agents targeting Hsp90 are under development. However, no promising candidates have been identified that effectively target the central nervous system. Therefore, the development of an Hsp90-specific PET tracer for quantifying Hsp90 expression in the brain would offer a potential biomarker for characterizing neurodegenerative diseases and facilitate advancement of Hsp90-targeted therapies. Of the PET tracers targeting Hsp90 that have been developed to date, only a limited number are capable of penetrating the BBB. Cools and colleagues reported the ^11^C-labeling and biological evaluation of HSP990, contributing to the limited pool of brain-penetrating PET tracers targeting Hsp90 (Cools et al. 2025a). HSP990 has exhibited saturable binding to Hsp90 in both in vitro and in vivo evaluation systems. Attenuated Hsp90 expression, as indicated by HSP990 binding, has been observed in the brains of aged rodents, mouse brains with Alzheimer’s disease (AD), and human AD brain sections. PET imaging in rodent models also demonstrated reduced Hsp90 expression in aging and AD. However, HSP990 binds to Hsp90 expressed in circulating natural killer cells, and equilibrium brain uptake was not achieved within the ^11^C imaging time window in nonhuman primates, suggesting its unsuitability for quantitative analysis. To address this, fluoroethyl derivatives have been evaluated as ^18^F analogues (Cools et al. 2025b), but these showed reduced affinity for Hsp90, indicating the need for further development of ^18^F-labeled tracers.

### PET and SPECT tracer development via Copper-mediated radiohalogenation of divergent and stable aryl-boronic esters. Another step forward!

By Philip Elsinga.

In recent years copper-mediated radiohalogenation methodology has been successfully applied in the production of ^18^F-, ^123^I-, ^76/77^Br- and ^211^At-labelled radiopharmaceuticals. Especially, aryl boronic tetramethyl ethylene glycol ester precursors (precursor with BPin) have enabled rapid late-stage radiohalogenations. A good example is the production of [^18^F]FDOPA (Mossine et al. 2020).

Despite these successes, this production method suffers from several challenges with respect to the synthesis of the precursors because of their instability, i.e. easy hydrolysis of the BPin leaving group. This instability causes problems in silica-based purification of the BPin-precursors as well as decomposition during radiolabelling under basic or acidic conditions (proto- or hydroxy-deborylation) resulting in lower RCCs and more challenging purification by HPLC.

Therefore more stable precursors with boronic tetra-ethyl ethylene glycol ester leaving groups (EPin) and boronic tetra-propyl ethylene glycol ester (PPin) analogs of the BPin precursors were developed and evaluated anticipating their increased stability and reactivity (Craig et al. 2025).

Stability on TLC-plates and in aqueous acetonitrile was evaluated. Whereas EPin and PPin compounds showed to be stable on TLC silica, the corresponding BPin compound displayed multiple spots indicating degradation of the compound. A similar trend was found with respect to stability in MeCN/water (1/1). After 20 min of incubation, 40% of the BPin was degraded whereas the EPin and PPin compounds remained intact. Regarding radiohalogenation, RCCs for EPin and PPin were almost similar.

The authors developed a robust method for preparing radiohalogenated radiopharmaceuticals for both diagnostic and therapeutic purposes. Similar findings with EPin precursors were also recently reported by the Gouverneur group (Hadjipaschalis et al. 2025). The enhanced stability of the EPin and PPin groups offers greater flexibility in synthesizing precursors for Cu-mediated radiolabeling.

### Reduce kidney reabsorption with para-aminohippurate

By Hua Yang.

Kidney toxicity is often the dose-limiting factor in peptide- or small molecule-based radioligand therapy using radionuclides such as ^177^Lu, ^225^Ac, and ^212^Pb, due to the high renal uptake of these radiopharmaceuticals. Coinfusion of lysine and/or arginine has been employed to reduce renal reabsorption; however, its efficacy is limited in certain cases, and side effects such as nausea and hyperkalemia have been reported.

As an alternative, para-aminohippurate (PAH) has recently been evaluated in clinical studies and demonstrated comparable nephroprotective efficacy to amino acids in patients undergoing [^177^Lu]Lu-DOTA-TOC treatment. Clinically, PAH is used to measure effective renal plasma flow and acts as a substrate for organic anion transporters.

In a related study (Meckel et al. 2025) the authors compared PAH with amino acids for nephroprotection across various radiopharmaceuticals, including [^177^Lu]Lu-DOTA-RGD, [^177^Lu]Lu-DOTA-JR11, [^177^Lu]Lu-DOTA-TATE, [^177^Lu]Lu-DOTA-TOC, [^177^Lu]Lu-DOTA-sargastrin, [^177^Lu]Lu-PSMA-I&T, [^177^Lu]Lu-PSMA-11, and two recombinant proteins: [^177^Lu]Lu-DOTA-affiline-22 and [^99m^Tc]Tc-etarfolatide.

The study found that PAH significantly reduced renal uptake of [^177^Lu]Lu-DOTA-RGD, [^177^Lu]Lu-DOTA-JR11, [^177^Lu]Lu-DOTA-TATE, and [^177^Lu]Lu-DOTA-TOC. Kidney protection was moderate with [^177^Lu]Lu-DOTA-sargastrin and less pronounced with the PSMA-targeting ligands. No reduction in renal uptake was observed with the larger recombinant proteins.

Overall, this study highlights the potential of PAH to reduce kidney uptake for select small molecule or peptide-based radiopharmaceuticals, emphasizing the importance of case-specific selection of nephroprotective agents.

### Precise radiopharmaceutical therapy: shaping patient futures with digital twins

By Klaus Kopka.

Radiopharmaceutical therapy (RPT) holds immense promise for cancer treatment, yet its current “one-size-fits-all” approach results in inconsistent patient outcomes due to a lack of personalization. To overcome this, the paradigm of Computational Nuclear Oncology (CNO) is proposed (Yusufaly et al. 2025), centered on Theranostic Digital Twins (TDTs).

**Fig. 4:**
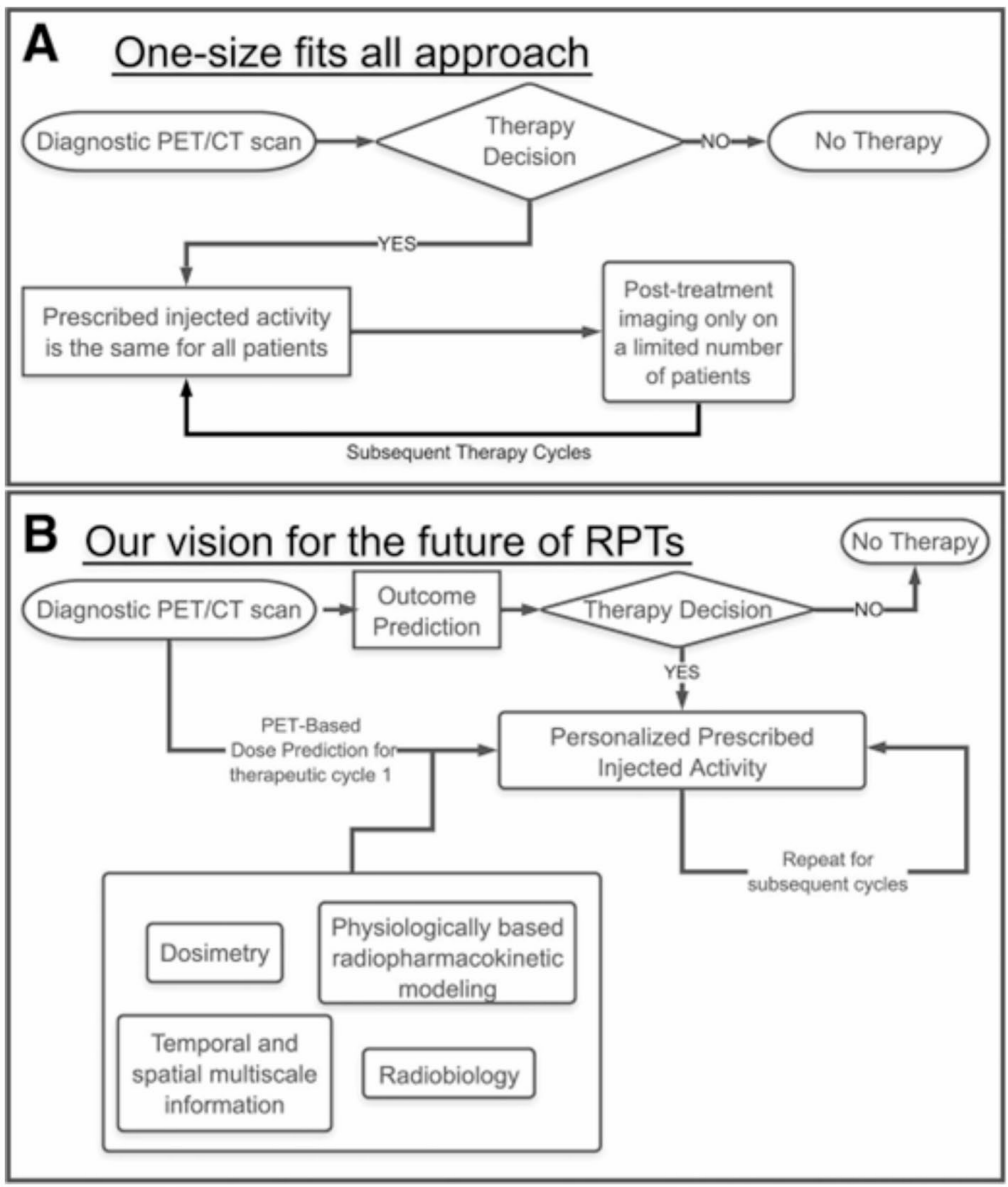
Diagram comparing present “one-size-fits-all” RPT approach (**A**) with vision of precision RPTs (**B**). Central to such a future is the development of robust models that translate therapeutic inputs to clinical outputs, from which one can prescribe optimal protocols in cycle 1 and refine the process in later cycles to adaptively optimize treatment. (This diagram was originally published in JNM. Yusufaly T et al. Computational Nuclear Oncology Toward Precision Radiopharmaceutical Therapies: Current Tools, Techniques, and Uncharted Territories. J Nucl Med. 2025;66:509–515. ©SNMMI)

A TDT is a virtual, patient-specific avatar, parameterized with pre-therapeutic diagnostic imaging and clinical data. These digital twins prospectively predict dosimetry and outcomes, enabling the optimization of individual activity prescriptions and dynamic adjustments to treatment plans throughout the therapy cycle, as vividly illustrated in Fig. 4. This dynamic capability allows for real-time monitoring, simulation of various treatment scenarios, and prediction of results, moving RPT towards a truly adaptive approach.

Successful implementation of CNO-guided TDTs requires robust aggregation and integration of heterogeneous data across multi-institutional cohorts, necessitating harmonized data collection and modeling protocols. While ethical and legislative concerns regarding data sharing pose practical hurdles, solutions like federated learning are being explored to ensure patient privacy while enabling collaborative research.

Ultimately, CNO powered by TDTs, offers a transformative shift for RPT. By integrating comprehensive patient data, clinicians can optimize therapeutic efficacy and minimize toxicity. This framework promises a future where treatment decisions are guided by predictive capabilities, leading to improved efficacy and reduced side effects, fundamentally enhancing the patient experience. CNO also fosters cross-institutional data sharing and intermodal collaboration, seamlessly integrating RPT into broader computational oncology efforts. This framework is poised to realize the long-term vision of truly adaptive and personalized cancer treatment.

### Radiolysis impact of ^211^At in its own radiochemistry

By Mickaël Bourgeois.

Astatine-211 (^211^At) is a highly promising radionuclide for targeted alpha therapy (TAT). However, there is no stable isotope of astatine meaning only the radioactive trace can be studied and, to date, its chemistry remains poorly understood. ^211^At is produced by a cyclotron nuclear reaction and isolated by dry distillation (^211^At vapors are classically condensed in chloroform).

In the highlighted article it has been investigated how the radiochemical yield (RCY) of the most two common astatine radiolabeling reactions (electrophilic destannylation of an organotin reagent and nucleophilic substitution on an iodonium salt) could be impacted by radiolysis associated with astatine-211 decay (Hansson et al. 2025). At high precursor concentration, RCY remained > 80% even after 28 h of decay. In contrast, at low precursor concentration, RCY dropped sharply over time regardless of astatine storage form (chloroformic solution or dry form obtained after chloroform evaporation). Electrophilic destannylation appears to be more sensitive to radiolysis effect, likely due to the chloroform radiolysis (i.e. formation of HCl, Cl_2_ and different halocarbons compounds). ^211^At speciation appears to be modified by the radiolysis in chloroform, but this phenomenon could be inverted by the re-dissolving of ^211^At in fresh chloroform or by adding an oxidant such as N-chlorosuccinimide.

This publication highlights the importance of time to optimize the ^211^At RCY, especially for applications which require high specific activity such biomolecules (peptide, monoclonal antibodies and their derivatives) radiolabeling.

### Chasing the daughters: tracking unchelated ²²¹Fr and ²¹³Bi from ²²⁵Ac recoil in TAT

By Frederik Cleeren.

Targeted alpha therapy (TAT) leveraging actinium-225 (^225^Ac) offers exceptional therapeutic efficacy due to the high linear energy transfer (LET) of its alpha emissions, especially when used in combination with prostate-specific membrane antigen (PSMA) targeting radioligands. Nonetheless, the clinical translation of ^225^Ac-based radiopharmaceuticals is hindered by the physicochemical behavior of recoil daughter radionuclides, particularly francium-221 (^221^Fr) and bismuth-213 (^213^Bi). The release and systemic redistribution of these recoil daughters following alpha decay can lead to significant off-target irradiation and toxicity, complicating dosimetry and safety profiles.

Zitzmann-Kolbe et al. systematically investigated the biodistribution of free ^221^Fr and its decay product ^213^Bi in LNCaP tumor bearing mice (Zitzmann-Kolbe et al. 2025). Their findings reveal rapid systemic clearance of both radionuclides, with ^221^Fr accumulating notably in kidneys, salivary glands, and intestines, while ^213^Bi showed preferential retention in kidneys and liver. Strikingly, ^221^Fr exhibited over threefold higher uptake in salivary glands compared to ^213^Bi, highlighting distinct organ-specific affinities. This supports the idea that free recoil daughters contribute significantly to off-target toxicity observed in TAT, especially in sensitive tissues like the salivary glands.

By separating ^221^Fr and ^213^Bi from their ^225^Ac parent prior to injection, the authors eliminate confounding in vivo generation effects, enabling clearer and more accurate insights into the redistribution of daughter radionuclides. These findings underscore the urgent need to incorporate recoil daughter kinetics into dosimetry and toxicity models for targeted alpha therapy (TAT). However, the use of ^225^Ac-labeled compounds with varying pharmacokinetic profiles will inherently affect the spatial and temporal distribution of free ^221^Fr and ^213^Bi, contingent upon the specific organs where these recoil daughters are generated. Consequently, the biodistribution and re-localization of these daughter radionuclides must be interpreted on a compound-by-compound basis to accurately assess their impact on both therapeutic efficacy and off-target toxicity. Overall, this work sets a new benchmark for the preclinical evaluation of alpha emitter decay chains and will play a critical role in guiding the safer and more effective design of ^225^Ac-based radiopharmaceuticals for clinical use.

### Will lead-212 generators move forward the targeted alpha therapy?

By Sergio Todde.

Targeted alpha therapy is showing an enormous interest, due to the potentially ideal properties of α-emitters in the treatment of tumours. Lead-212 is a β^−^ -emitting radionuclide that generates, by decay, the α-emitter ^212^Bi with a suitable half-life of 10.6 h. The parent nuclide is ^228^Th, which is now available from various sources, that ultimately led to the marketing of generators capable to reliably provide clinical doses of ^212^Pb via automated and safe procedures. The highlighted article comprehensively describes the state of the art in R&D of ^212^Pb chemistry, discussing the various bifunctional chelators and conjugation strategies, as well as the most significant clinical trials currently in progress and related findings (Scaffidi-Muta & Abell 2025). In general, ^212^Pb matches the behaviour of other known α-emitters such as ^225^Ac, with similar pros and cons, but also with its own specific characteristics. Major cons are represented by the high rate of dissociation of the radionuclide from the chelator-vector conjugate, that may lead to significant radiotoxicity caused by e.g. kidney accumulation, and by the difficulty to correctly evaluate dosimetry, for which suitable imaging protocols are not yet fully available. Indeed, even the use of genuine lead radionuclides such as ^203^Pb do not fully match the complicated decay scheme and the dissociation of the intended radionuclide, thus not allowing for an accurate dosimetric profile. On the other hand, strategies to overcome or mitigate the above problems are continuously improved, and the availability of a broad arsenal of suitable chelators and radiolabelling strategies may lead to a significant step forward in the clinical use of this interesting radionuclide.

### Two targets, one mission: heterobivalent metal-based radiopharmaceuticals for prostate cancer therapy

By Silvio Aime.

The highlighted article is an interesting, timely review covering the numerous efforts that have ben carried out in recent years to improve the efficiency of metal-based radiopharmaceuticals for prostate cancer (PCa) imaging and therapy (Sobral et al., 2025). In this field much attention has been gathered by [^177^Lu]Lu-PSMA-617 (Pluvicto), that has recently entered the clinical TRT practice for the treatment of mCRPC by targeting the PSMA receptor, which is significantly overexpressed in PCa lesions, while remaining minimally expressed in normal tissues.

**Fig. 5:**
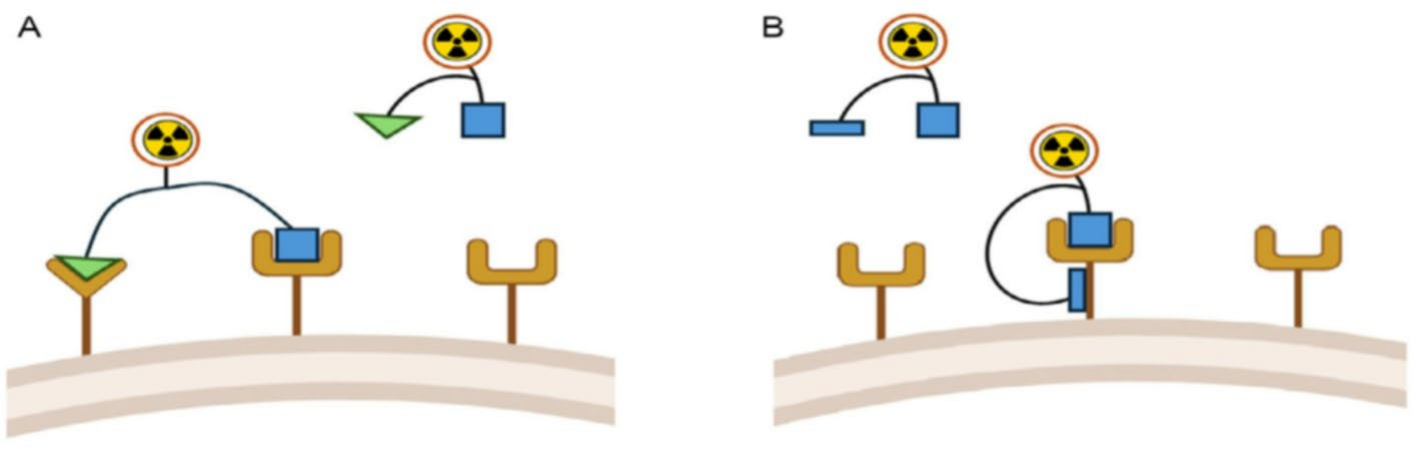
Schematic representation of two types of dual-target radiopharmaceuticals, i.e. the “heterobivalent” ones that interact with two distinct molecules (**A**) and the “bitopic” ones interacting to two sites on the same molecule (**B**). Figure reproduced from Sobral et al., 2025 under the CC BY license

The topic is particularly relevant in light of cancer resurgence in a non-negligeable fraction of treated patients showing PSMA-positive lesions. The acquired resistance appears as a result of mutations in the targeted proteins, and thus it appears necessary to develop strategies to improve efficacy and overcome resistance. There are two types of dual-target radiopharmaceuticals, i.e. the “heterobivalent” ones that interact with two distinct targets, such as separate receptors (A), and the “bitopic” ones interacting with two sites on the same receptor (B) (Fig. 5).

Among the possible innovative therapeutic options, much attention has been devoted to the design and testing of radiopharmaceuticals that target two proteins. The dual-target approach is expected to decrease the adaptive resistance, as this would typically require alterations in both targets. Actually, the simultaneous binding of the agent to two targets should lead to a synergistic increase in binding affinity. Moreover, they may provide useful insights into tumor heterogeneity. Beyond PSMA targeting (operated by the glutamate-urea-X vector, where X refers to lysine, cysteine, or another glutamate), the design of heterobivalent probes has included vectors targeting the gastrin-releasing peptide receptor (GRPR), integrin αvβ3 receptor, fibroblast activation protein (FAP), and sigma-1 receptor, as well as synthons able to bind to serum albumin to prolong the circulation lifetime.

When a theranostic pair is considered, one may realize how isostructural radioconjugates incorporating radionuclides of different elements may exhibit variations in biological distribution, uptake, or clearance. This can result in differences between the diagnostic imaging results and the actual therapeutic delivery, thus impacting the efficacy of the treatment. The achievements summarized in this review clearly indicate how minor variations in chemical structure can result in marked changes in biodistribution and targeting ability whose understanding will contribute to the design of improved agents for the field of TRT for PCa and other tumor types.

### Actinium-225 radiochemistry through the lens of [^225^Ac]Ac-DOTA-TATE

By Eduardo Savio.

Alpha particles have a higher linear energy transfer (LET) and a shorter tissue range (≤ 100 μm), causing higher probability of DNA double strand breaks with a reduced chance of damaging surrounding healthy cells, compared to beta particles. Actinium-225 is an alpha-emitting radionuclide considered particularly promising for targeted alpha therapy because of its favourable half-life (9.92 days) and its emission of four α-particles in the decay cascade. Actinium-225 might be an interesting candidate for small molecules in applications where a high tumor accumulation is possible. Peptide receptor radionuclide therapy was evaluated with different peptides, having the potential as an effective treatment modality, especially for cancers that are resistant to conventional care or if surgical options are limited. There is growing interest in optimizing the quality control (QC) methods and radiolabeling process for ^225^Ac. Currently, there are no European Pharmacopoeia (Ph. Eur.) ^225^Ac-based radiopharmaceutical monographs available.

This study determined critical parameters and established optimal labeling and accurate measuring techniques for radiochemical yield and purity with DOTA-TATE as a model molecule (Hooijman et al. 2025). ^225^Ac sources were analyzed for metals (ΣFe, Zn, Cu) and quantified by UPLC. Optimization of radiolabeling kinetics for clinical conditions was performed in regards to temperature (20–90 °C), heating time (5–60 min), pH (2.5–10, with/without excess of metal ions), buffers, quenchers, volume (0.1–10 mL) and molar activity (90–540 kBq/nmol). The quality control was investigated using radio-TLC/HPLC by changing gradient to evaluate peak separation, radiolysed peptide and impurity separation.

The purity of ^225^Ac-labeled DOTA-radiopharmaceuticals was shown to be dependent on the pH and composition of the reaction mixture. It was also shown that labelling with ^225^Ac at higher pH reduces the influence of metal impurities. Optimized radiolabeling conditions, accurate measurements and improved quality control methods were essential for achieving an optimal measurement of the radiochemical yield and radiochemical purity/stability. The authors concluded that optimization of these parameters is strongly recommended to ensure effective and safe patient treatment.

## Conclusions

Trends in radiochemistry and radiopharmacy are highlighted. Hot topics cover the entire scope of EJNMMI Radiopharmacy and Chemistry, demonstrating the progress in the research field in many aspects.

## Data Availability

Datasets mentioned in this article can be found in the cited articles.

## Abbreviations

AD: Alzheimer’s Disease
BBB: Blood Brain Barrier
CNO: Computational nuclear oncology
DC: Decay-corrected
DNA: Deoxyribo nucleic acid
EMA: European Medicines Agency
FAPI: Fibroblast Activating Protein inhibitor
FDA: Food and Drug Administration
GMP: Good Manufacturing Practice
Hsp90: Heat shock protein 90
HSP990: (R)-2-amino-7-(4-fuoro-2-(6-methoxypyridin-2-yl)phenyl)-4-methyl-7,8-dihydropyrido[4,3-d]pyrimidin-5(6 H)-one
iSTP: Instant Single Imaging Time Point
LET: Linear energy transfer
mCRPC: Metastatic castrate-resistant prostate cancer
PCa: Prostate cancer
PET: Positron emission tomography
PSMA: Prostate specific membrane antigen
RCC: Radiochemical Conversion
RCY: Radiochemical Yield
RPT: Radiopharmaceutical therapy
SPE: Solid phase extraction
TAT: Targeted alpha therapy
TDT: Theranostic digital twins
TRT: Targeted radionuclide therapy
